# Everybody's Page

**Published:** 1888-10-06

**Authors:** 


### Everybody's Page.

Sulphur is frequently the cause of skin diseases when it has been employed as a remedy for them.

Men who have to undergo severe mental or bodily exertion can bear a large amount of stimulus with comparatively little harm.

Baudelocque, a great French physician, speaking of air changed by respiration, remarks: "This is the true cause, the sole cause, perhaps, of the disease of scrofula."

Dr. Constantine Paul is of opinion that a scrofulous constitution is evidenced by the formation of a linear scar or slit-like opening where the lobule is pierced; but others have suggested that the weight of ear-rings may be sufficient to account for this peculiarity.

Loss of front teeth is a common cause of lisping. Congenital or acquired perforation of the hard palate, or destruction of the soft palate, causes the conversion of b into m, d into n, and hard g into ng, and gives more or less of a nasal quality to all the sounds.

Dental caries or decay of the teeth seems to be a process involving both vital and chemical action, some defective or morbid condition of the dental tissues leading to their being devitalised, and subsequently acted on by destructive and decomposing agencies of various kinds.

Work should not be demanded from a growing child immediately after food. The acts of digestion require a large supply of blood, and so long as these acts are in progress the rest of the system, and the brain in particular, must be comparatively bloodless, or if it be brought into play it diverts a certain quantity of the blood from its proper destination, and interferes with the due assimilation of the food.

A single drop of nicotine applied to the eye of a cat will kill the animal in a few minutes. A small bird will die from the inhalation of a portion of nicotine vapour so small as to be inappreciable by a fine balance. Rabbits, cats, and dogs, die in from twenty to thirty seconds with less than a drop placed on the tongue, so rapidly is it absorbed, and so violent are its effects; and man suffers severely with only the 25th of a grain.

In France, in 1833, as we learn from the reports furnished by Lorraine and the first visitor of schools appointed by M. Guizot, the school charges depended on whether the pupil was taught reading alone or reading and writing; and many children left school incapable of writing even their own names, and of those who could read some could only read print, whilst others could decipher writing. It is even said that in some cantons of Brittany, of the Limousin, and of Cevennes, there are schools where no writing is taught until the children can read easily and know their Catechism.

In America there are in every city numerous organizations under various names, such as "The St. John's Guild" and "Tribune Fresh-Air Fund" in New York, "The Poor Children's Excursions" in Boston, "The Children's Country Week" in Philadelphia, all of which take the children out for longer or shorter periods to the country. In many Transatlantic towns arrangements exist for giving young children sails on sea or lake during those hot months of summer which blast the infant lives of their population like the hot breath of the sirocco. Tickets for these sails are usually distributed through the Health Department or the Police, and the mothers accompany the children.

Probably the first man to demonstrate the existence of living particles in the air was Dr. Angus Smith, but others had previously rendered the fact extremely likely; and such were, first, the naturalist Redi, then Needham and Buffon, then Spallanzani, and later on Schwann and Schroeder and Von Duschn, who were able, by traps of cotton wool and other means, to intercept these particles, and devise means for the preservation of meat or of sugary juices.

It has been calculated that a single gas-jet with an ordinary burner produces, in the course of an hour, as much carbonic acid as seven adult human beings. To warm yourselves, therefore, by means of burning gas, without providing an effectual means of escape for the invisible smoke that it produces, is to expose yourselves to great risk of gradual deterioration of health, and often of immediate headache, from the noxious fumes that you take into your bodies.

The compass of the voice—that is, the range of musical notes which it can evolve—varies, in different persons, from one to three octaves, and its collective total range is about four octaves. The voices of men extend about an octave lower in the scale than those of women. But, on the other hand, women's voices reach about an octave higher than men's. The difference between the sexes in this respect is due to the fact that in men the larynx is larger than in women, and the vocal cords are longer.

Human beings are not singular in being affected by impure air. Many animals have been found to suffer from consumption when kept in close confinement. It is very prevalent amongst the cows which supply milk to the inhabitants of some large towns, where they are immured during part of every year in close dairies. This was at one time remarkably the case with the cows belonging to the milkmen of Paris, which were annually carried off by consumption in considerable numbers. Those who desire to pursue this subject are referred to the "Annales d'Hygiène," vol. xi. p. 447.

Cold in the head, the precursor of ear-disease, is often due to reckless sitting or standing in draughts. A man, after heating himself in running to catch a train, elects to grow cool by travelling with his face towards the full current of air from an open window, and very soon, in consequence, his friends have to commiserate with him upon a stiff neck or earache. Other and similar sources of danger to the ear are exposure to wet, damp feet, neglect to change the clothes after excessive perspiration, and cutting the hair too short, or washing it at bedtime.

In 1783 Dr. Joseph Clarke, then master of the Rotunda Hospital in Dublin, turned his attention to the ventilation of that institution. For twenty-five years there had been a most frightful mortality amongst the infants born in the hospital. In this period, in round numbers, out of 18,000 children born, no fewer than 3,000, or one in every six, had died, mostly within the first fortnight after birth, of what was commonly called "nine-day fits." This disease was a peculiar form of convulsion, affecting first the muscles of the lower jaw and then passing on to other parts of the body, until it destroyed life by a general spasm of the muscles of breathing. After a thorough ventilation of the wards had been carried out the number of these cases diminished at once in a surprising manner, and in the next twenty-eight years, out of 15,072 children born, only 550, or one in every 104, died in the hospital. Dr. McClintock, of Dublin, during his mastership, ending in 1861, caused further improvements to be made in the ventilation, and since then the occurrence of this disease has been almost unknown.

